# Supplementary figures and images for: A Family of Viral Satellites Manipulates Invading Virus Gene Expression and Can Affect Cholera Toxin Mobilization
Source: mSystems. 2020 Oct 13;5(5):e00358-20. doi: 10.1128/mSystems.00358-20 (PMC7567579; doi:10.1128/mSystems.00358-20)

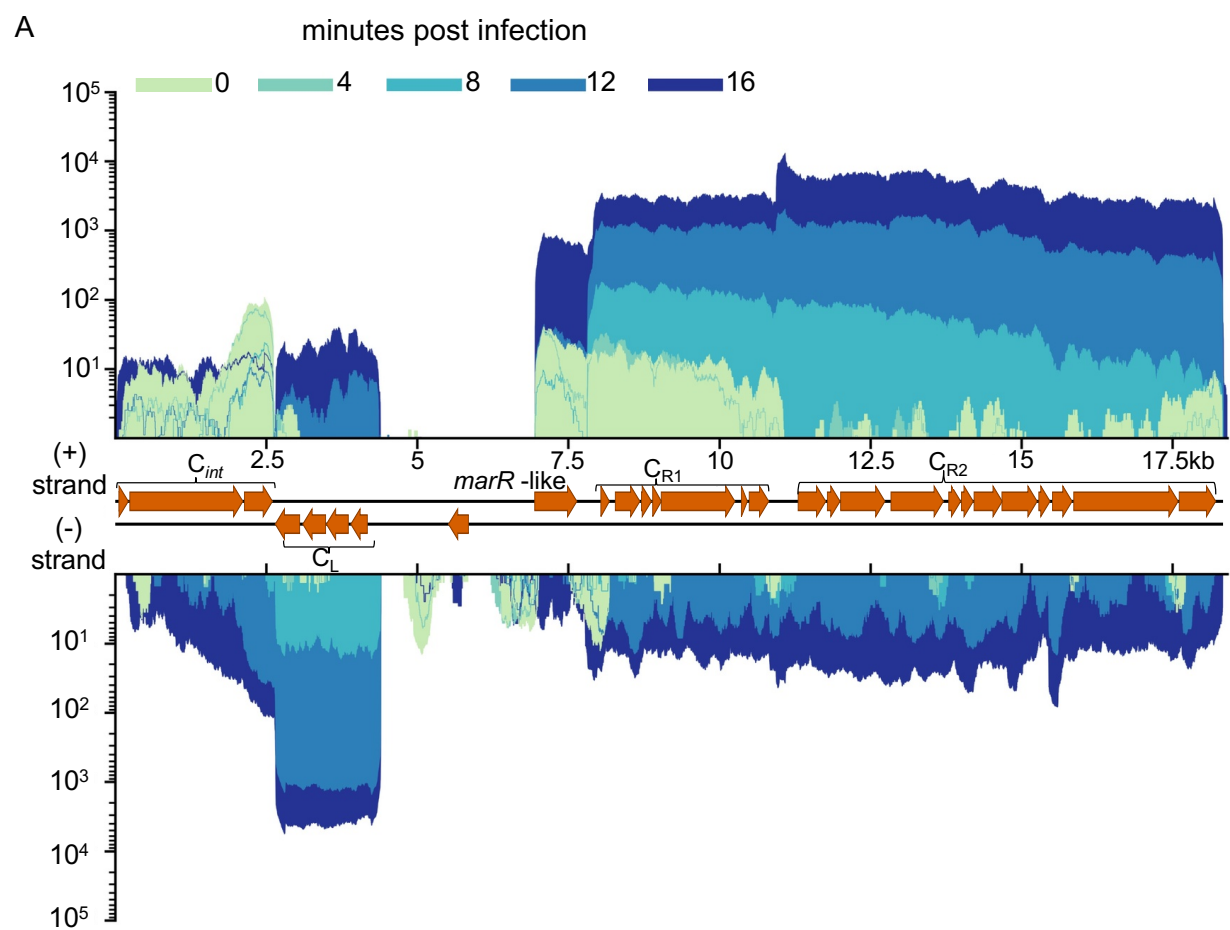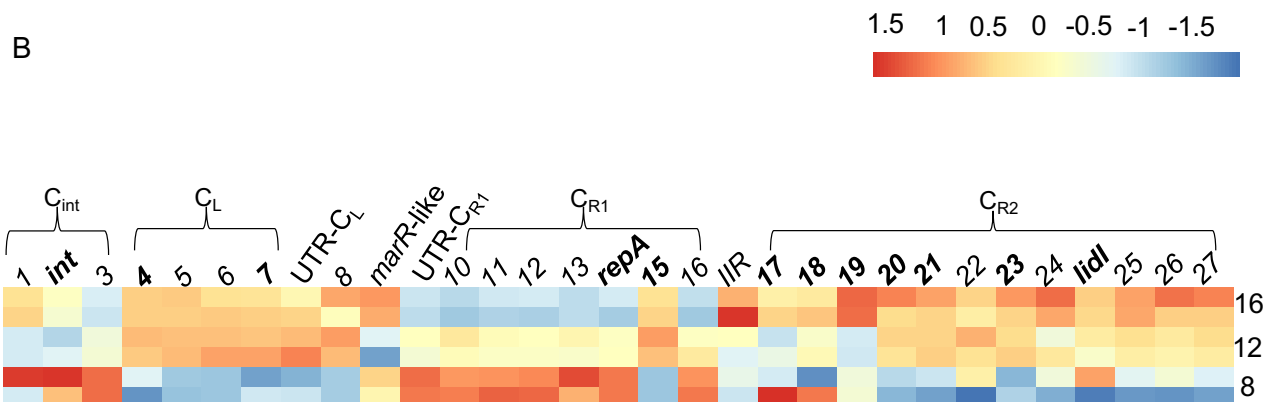

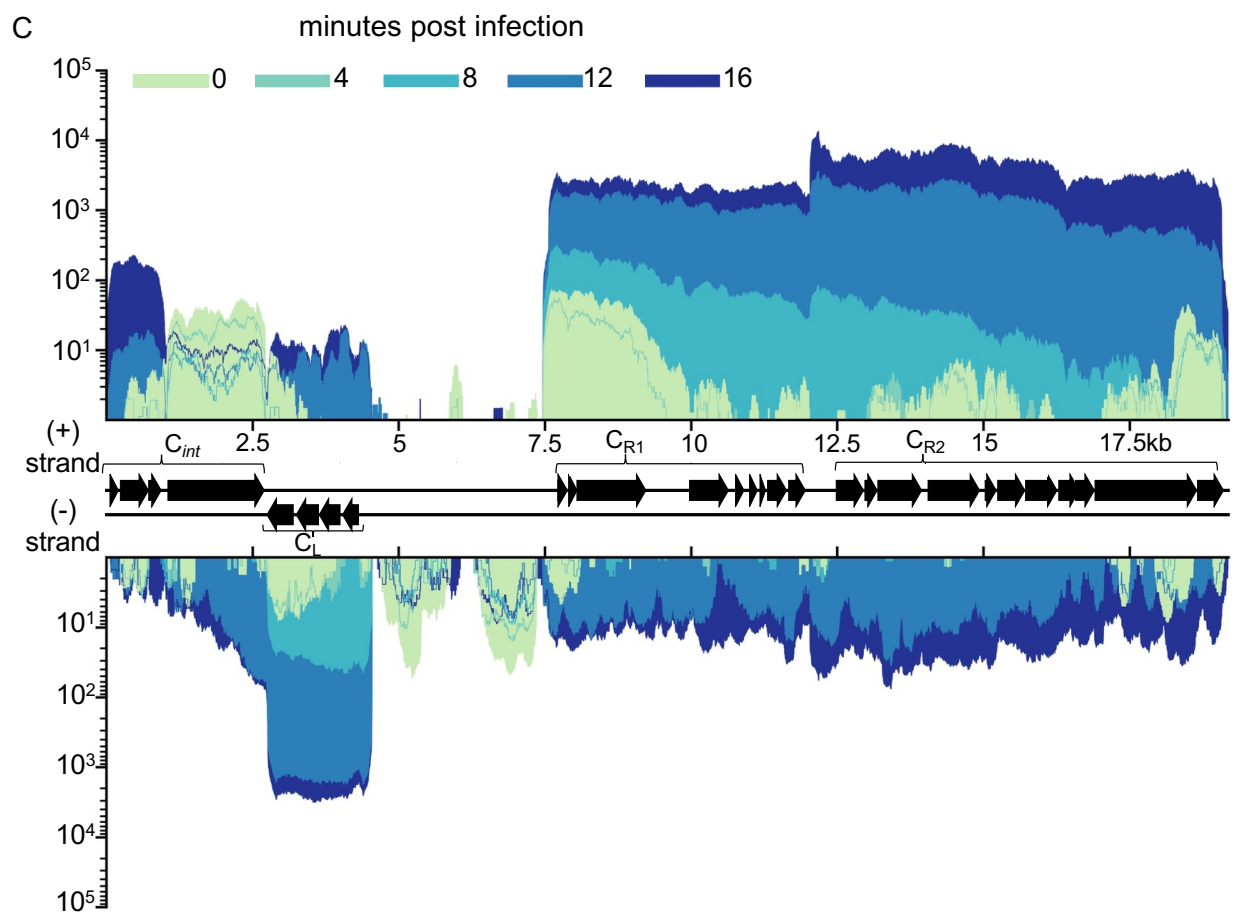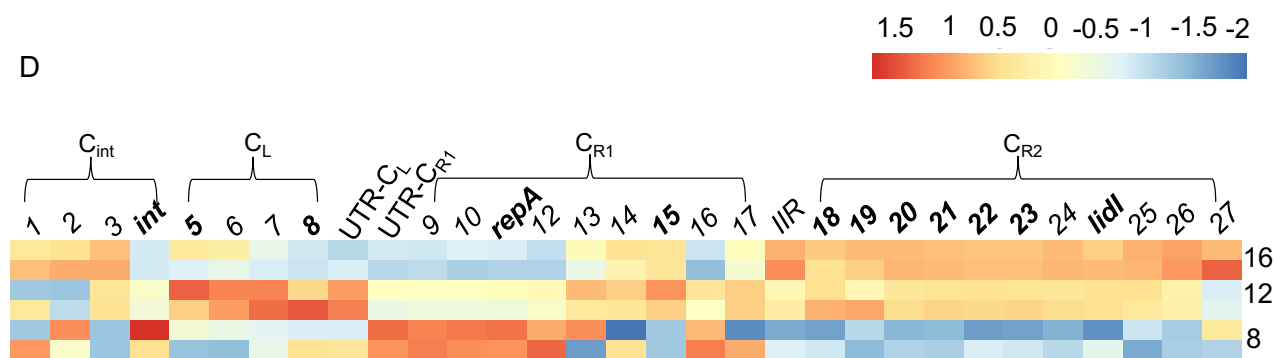

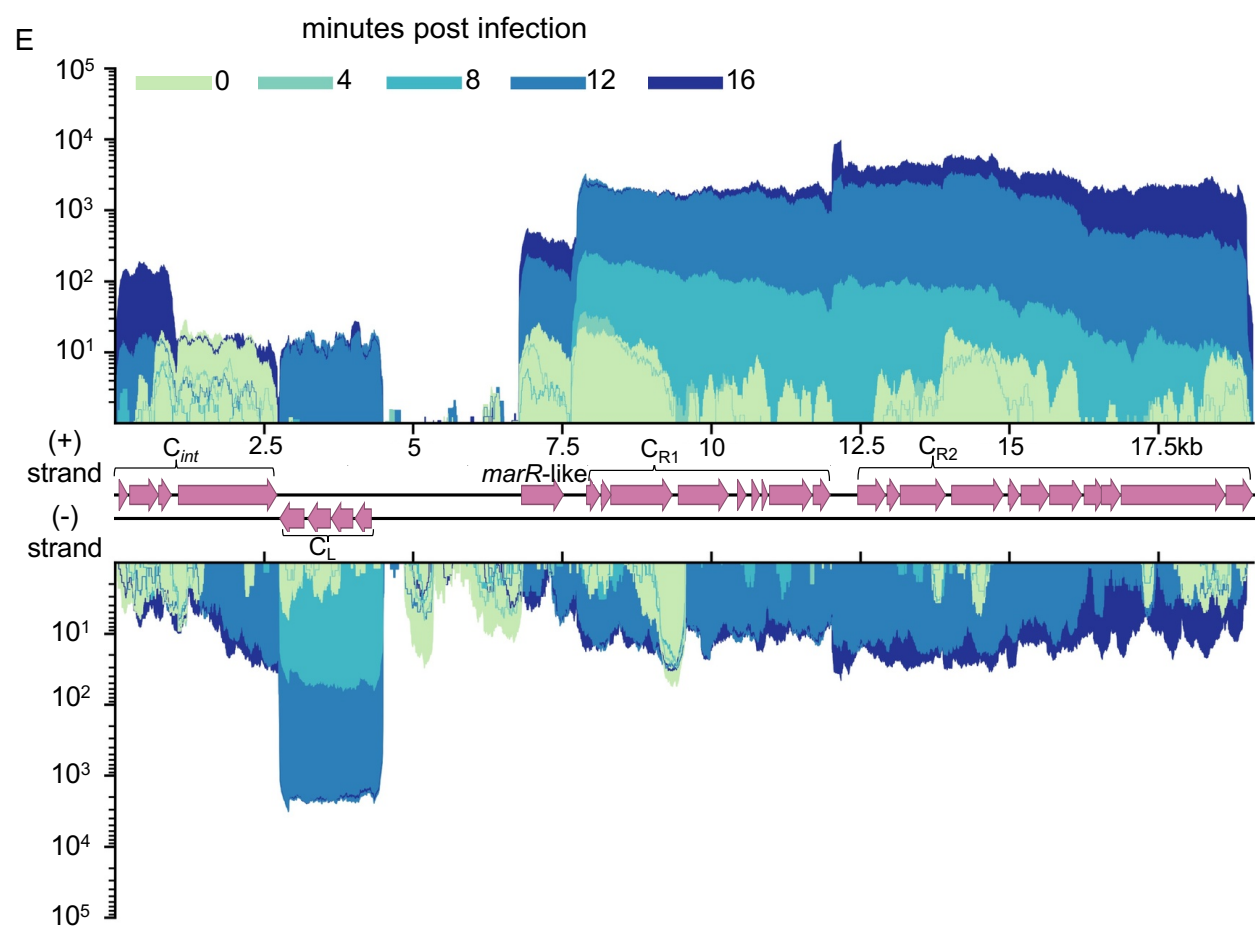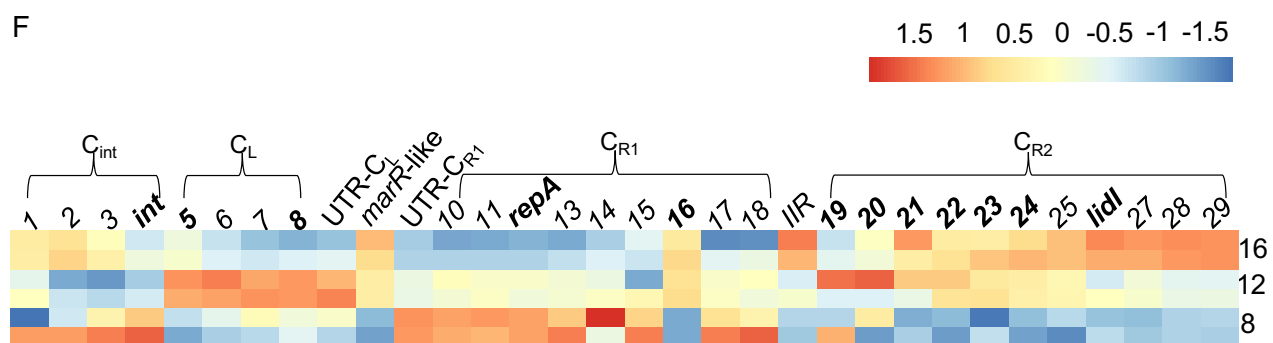

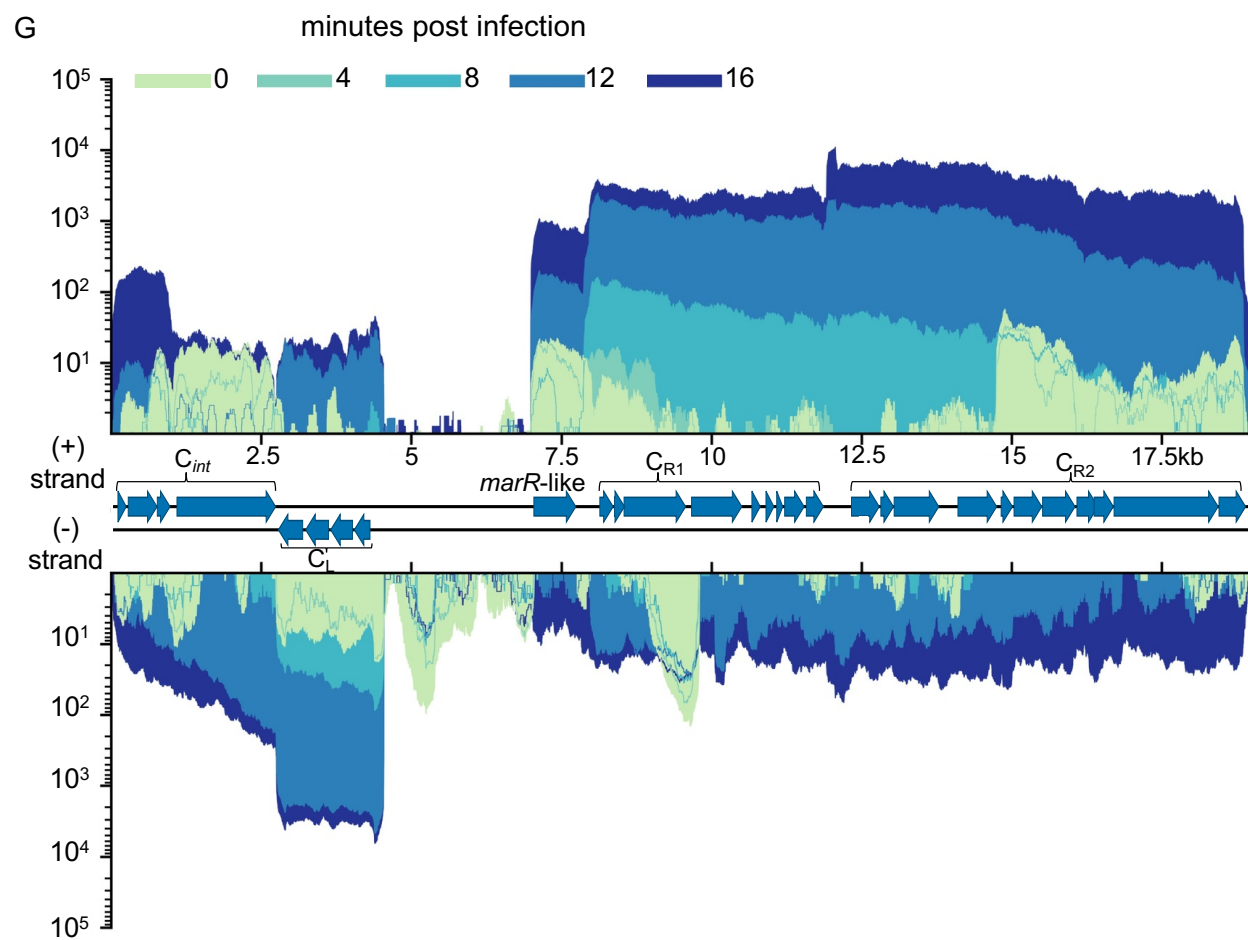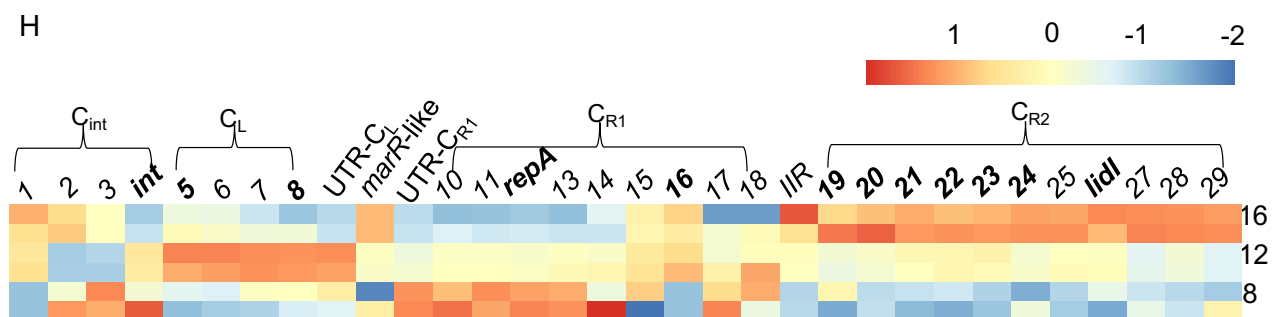

Supplement: FIG S1 [file mSystems.00358-20-sf001.pdf]

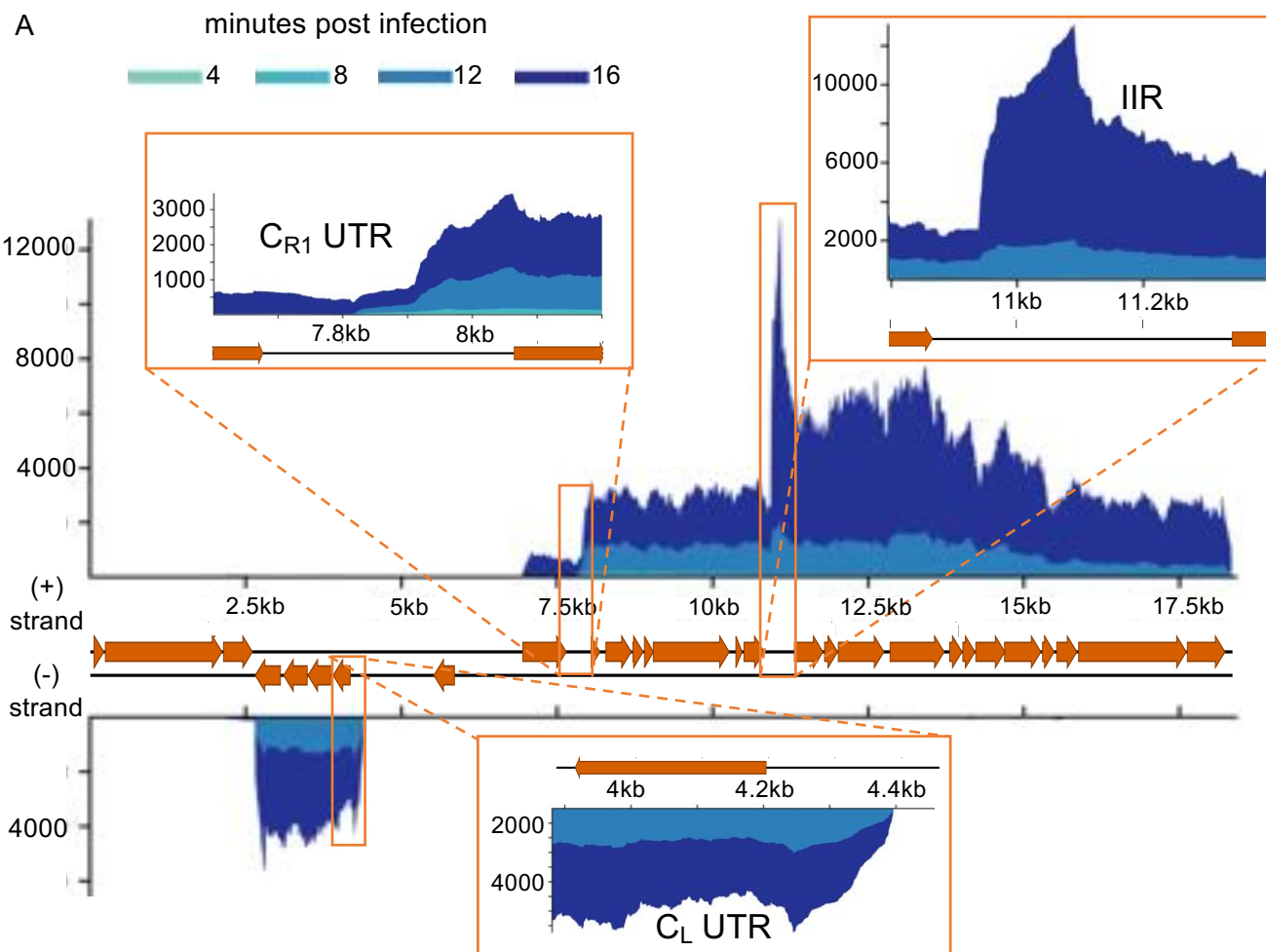

B

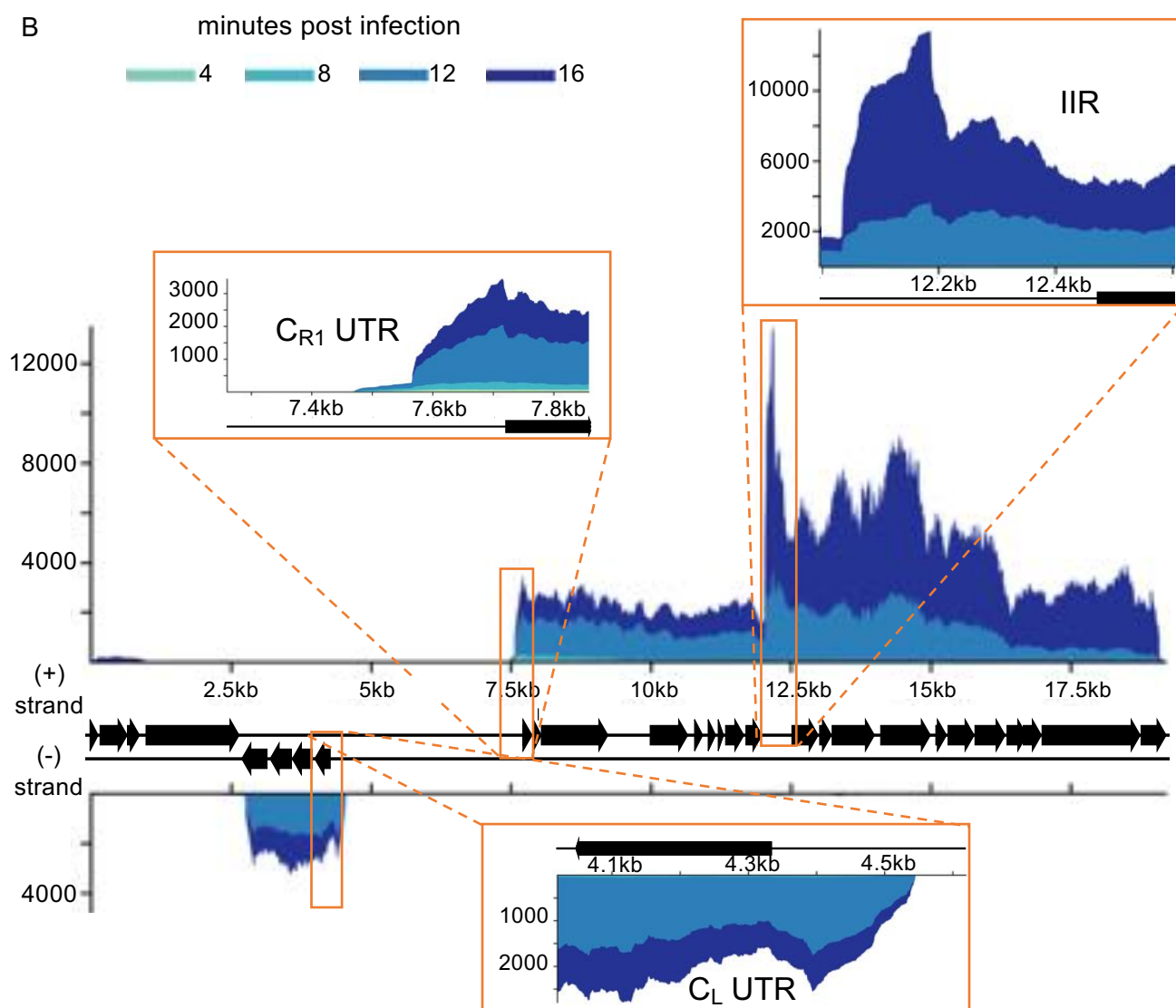

C

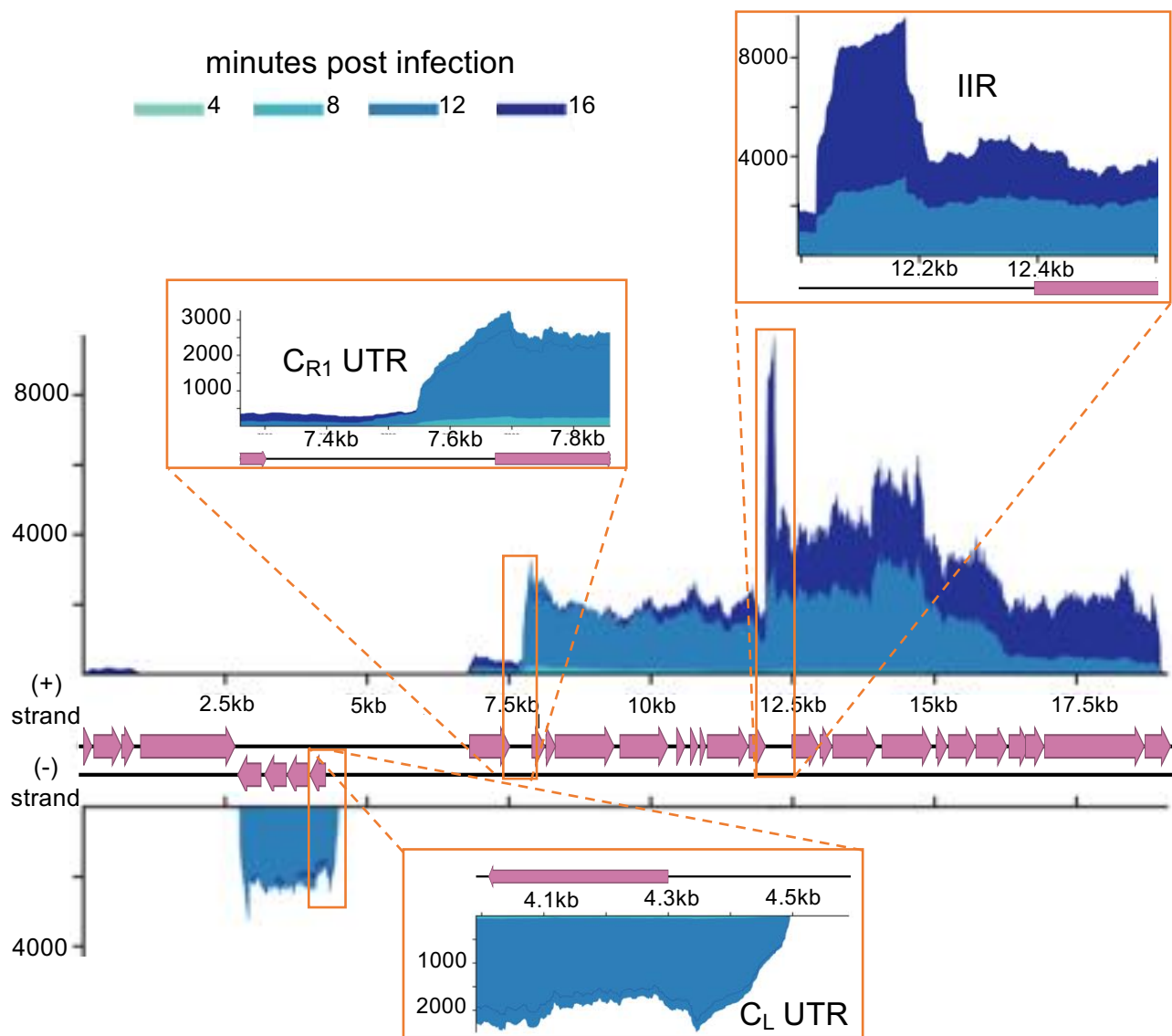

D

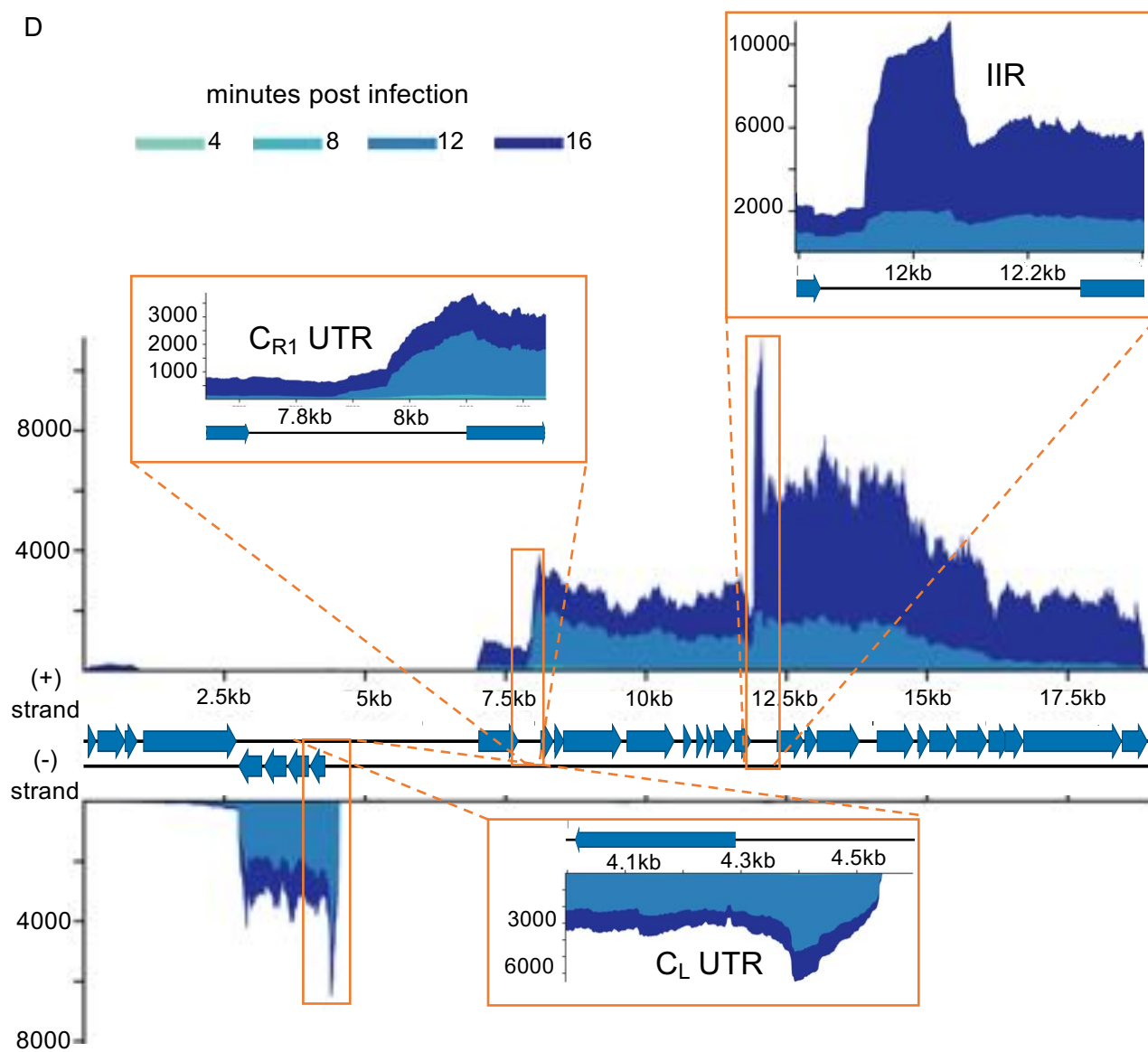

Supplement: FIG S2 [file mSystems.00358-20-sf002.pdf]

● IMEX encoded gene   ● Gene proximal to PLE   ● Other Superintegron gene   ● Other significant gene

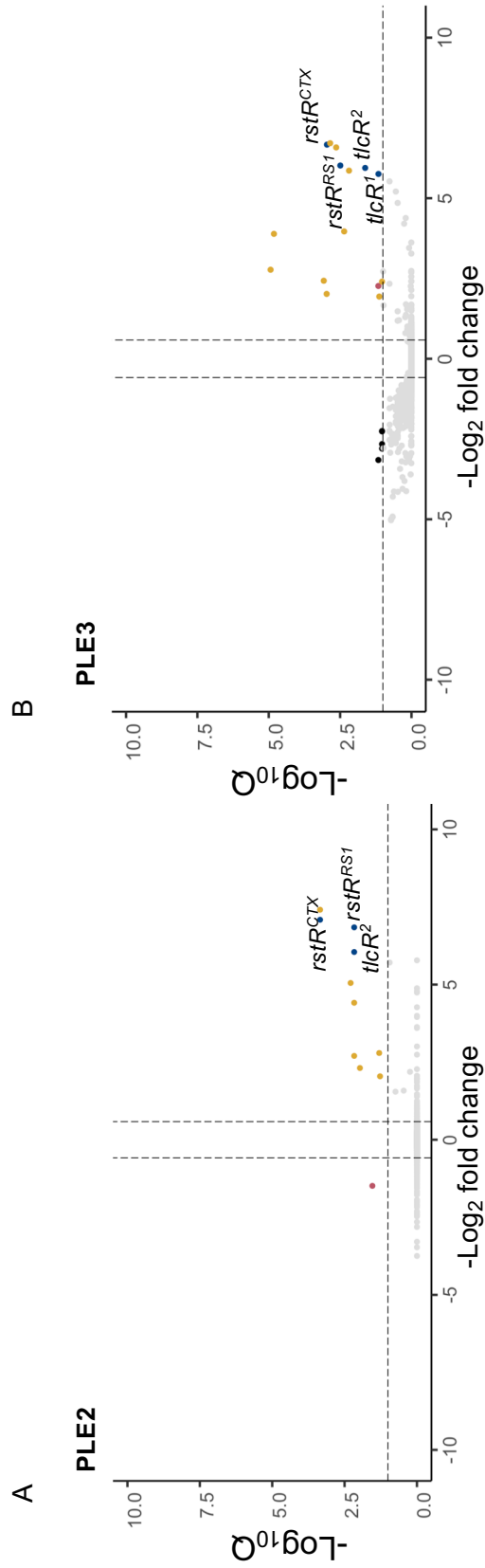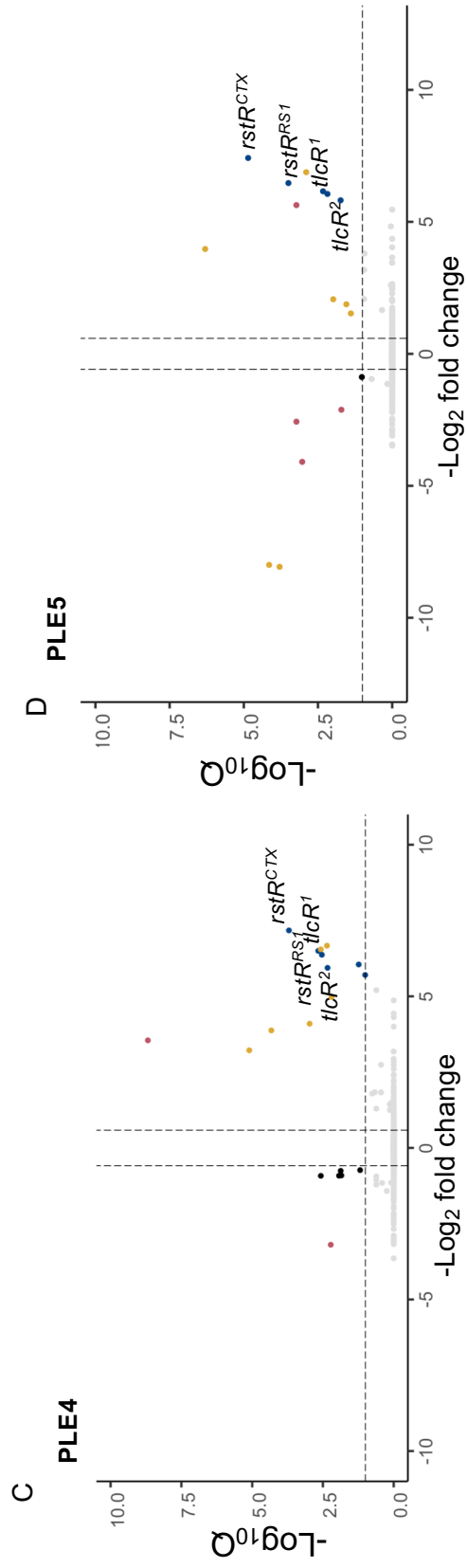

E

2kb

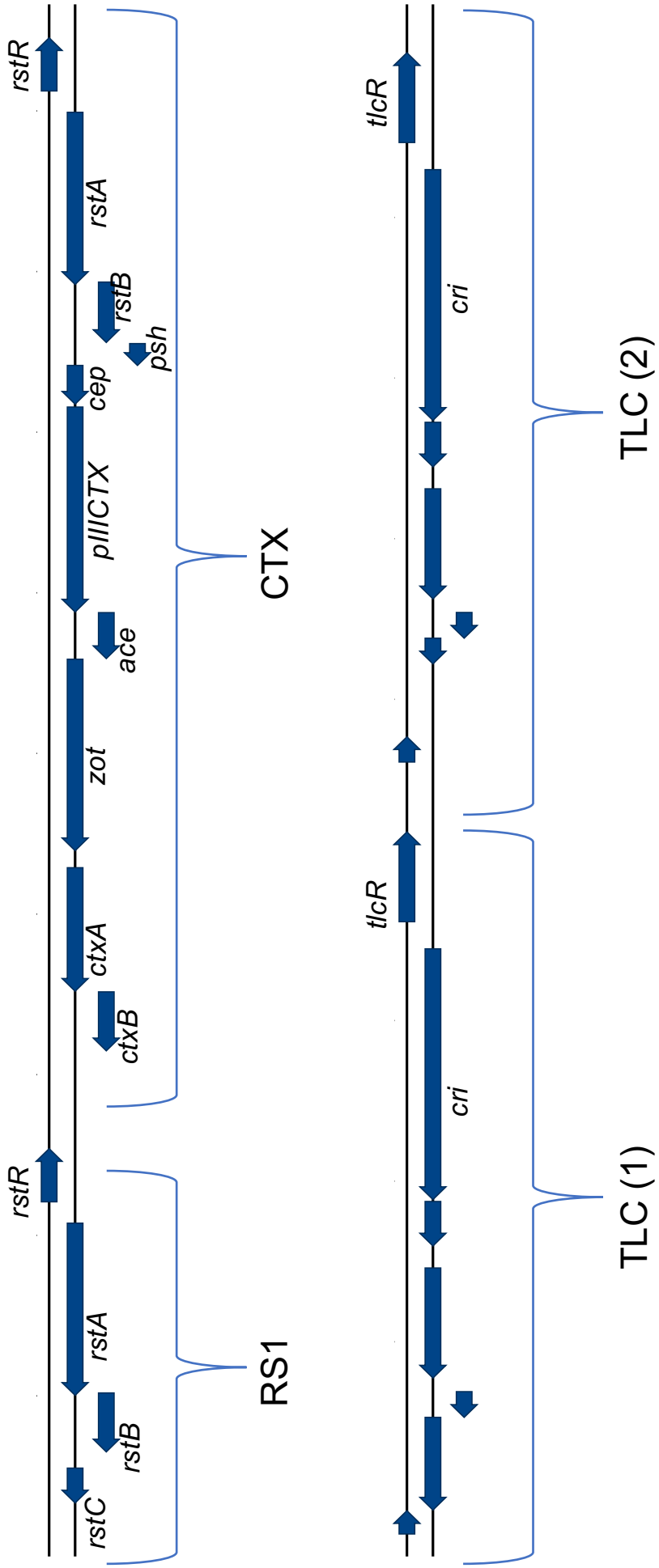

Supplement: FIG S4 [file mSystems.00358-20-sf004.pdf]

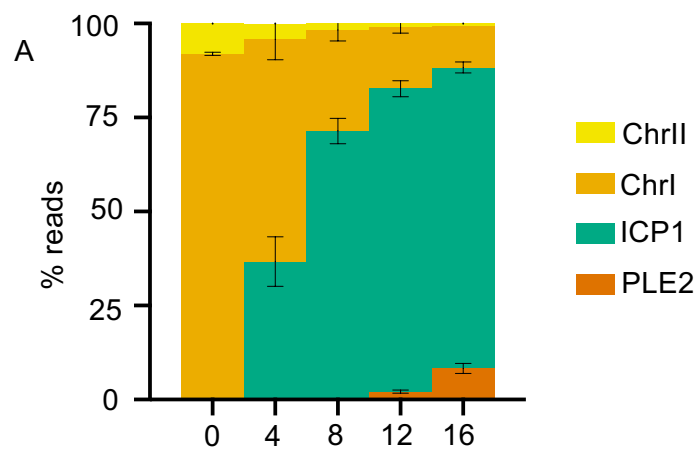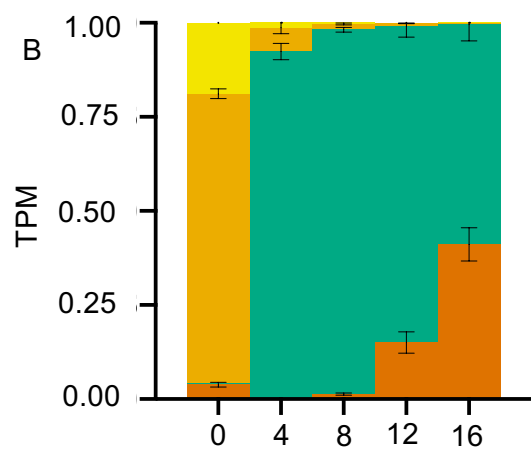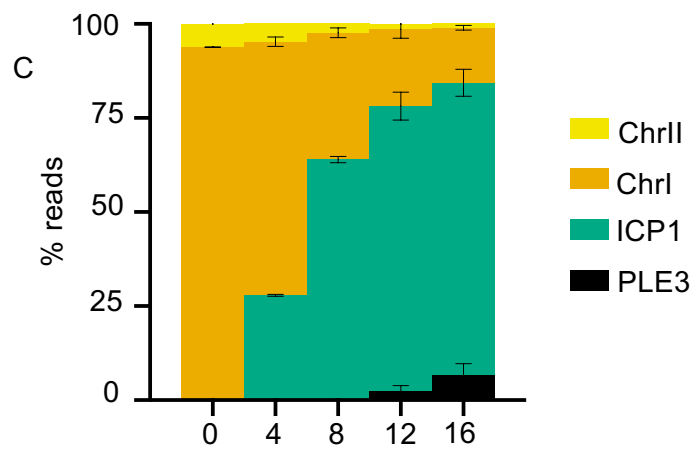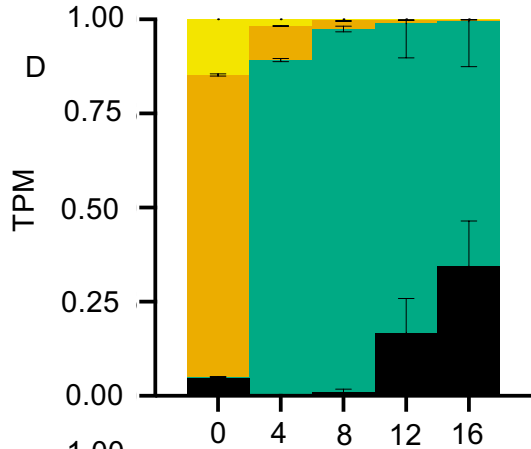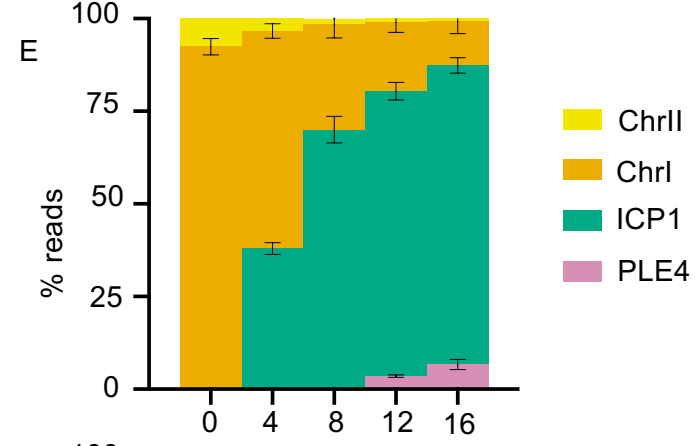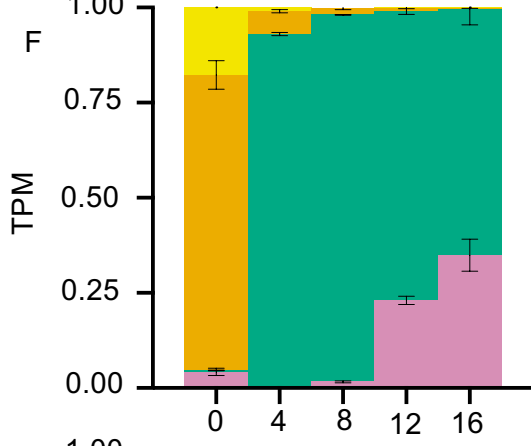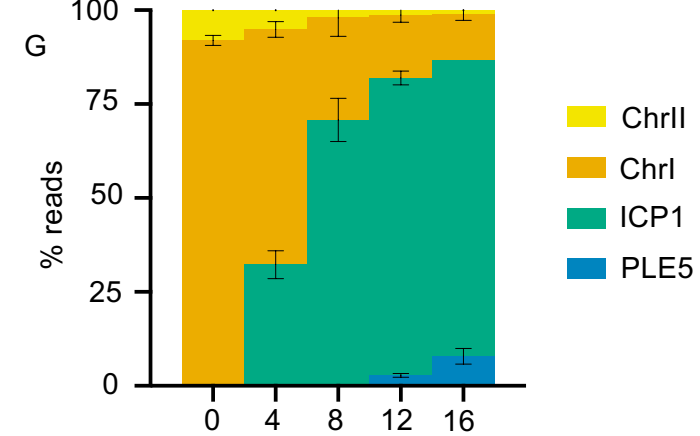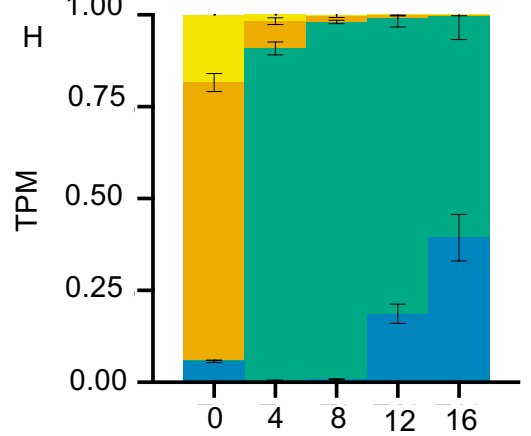

Supplement: FIG S5 [file mSystems.00358-20-sf005.pdf]

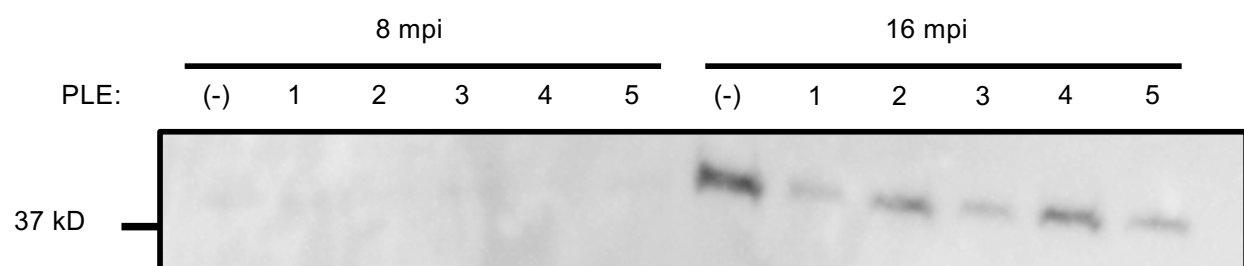

Supplement: FIG S6 [file mSystems.00358-20-sf006.pdf]

A

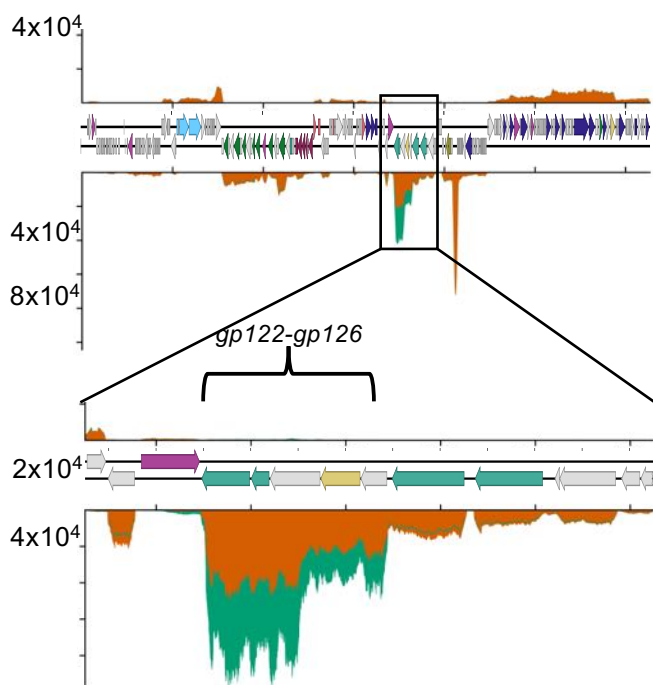

B

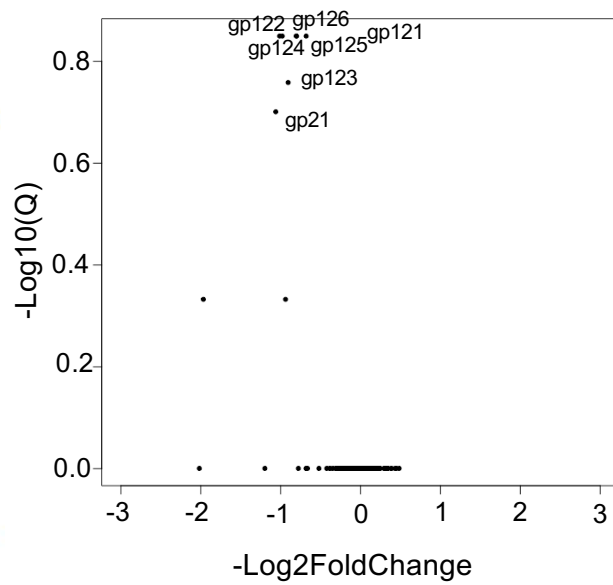

C

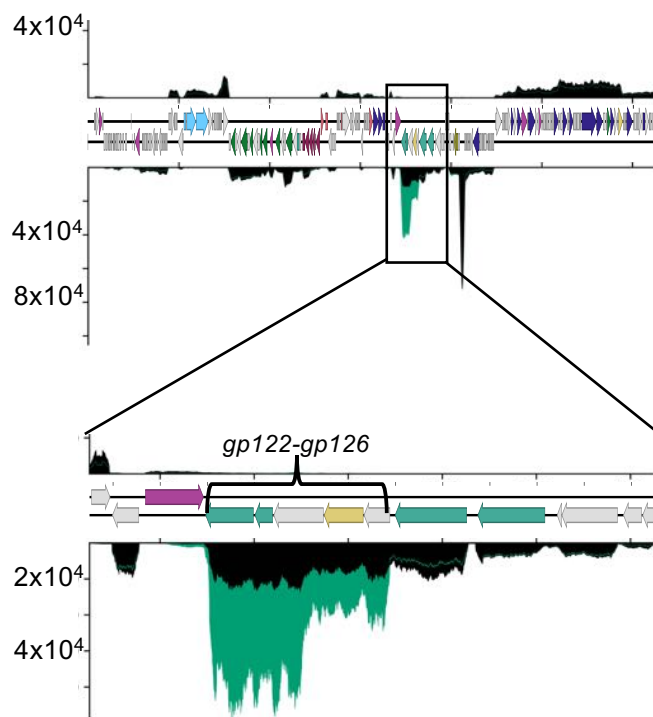

D

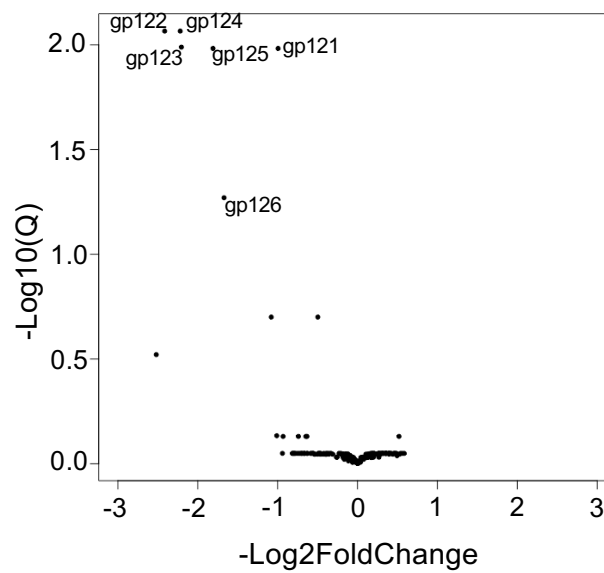

E

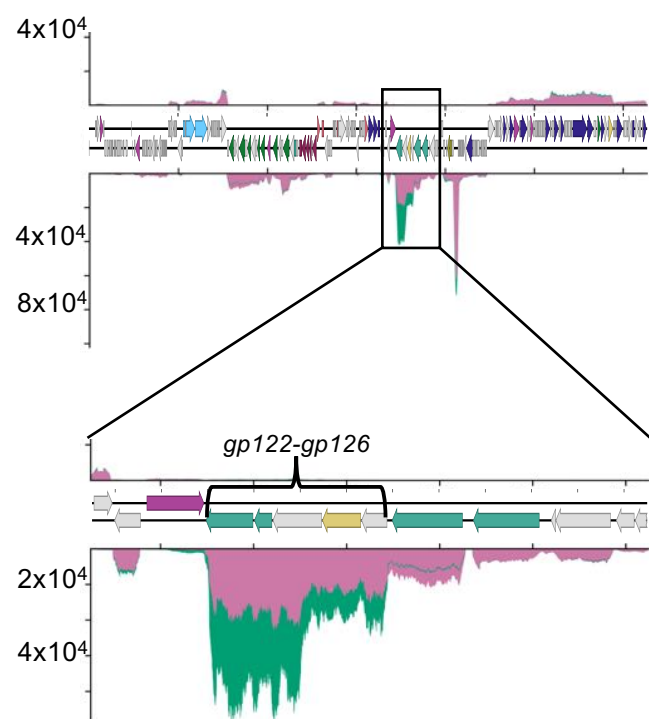

F

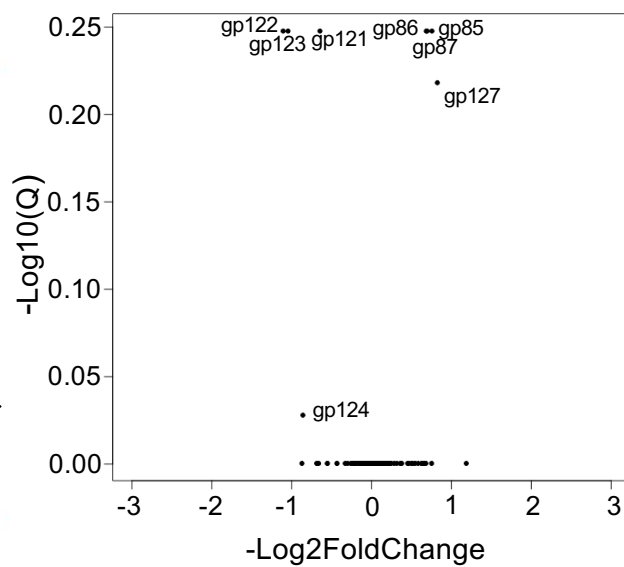

G

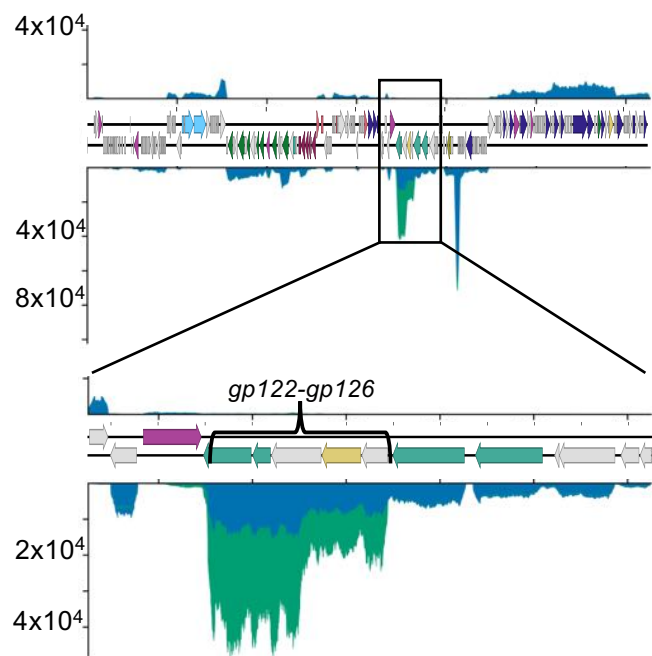

H

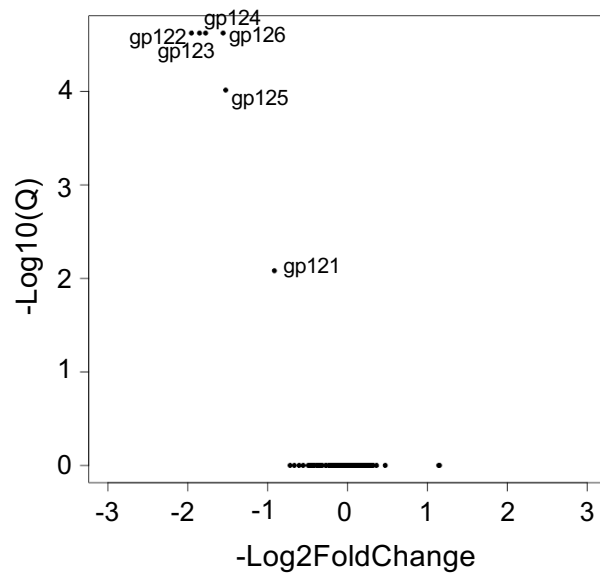

Supplement: FIG S7 [file mSystems.00358-20-sf007.pdf]

A

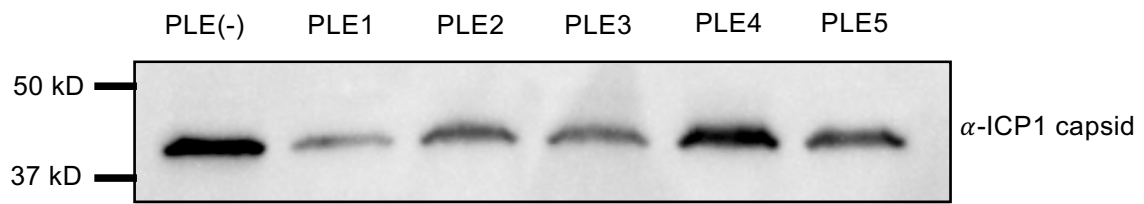

Standardized total protein 16 minutes post-ICP1 infection MOI=1

B

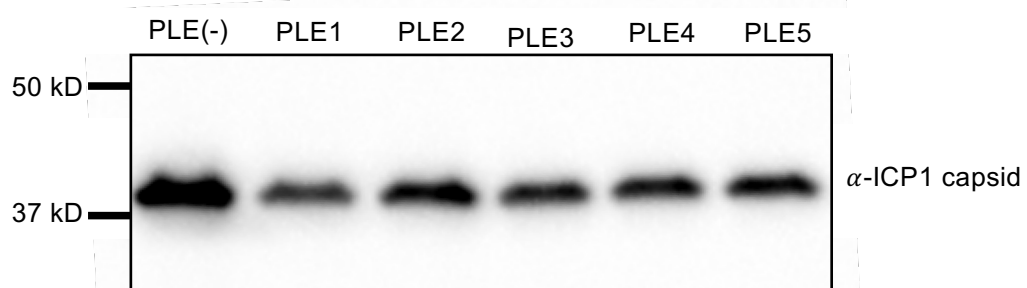

C

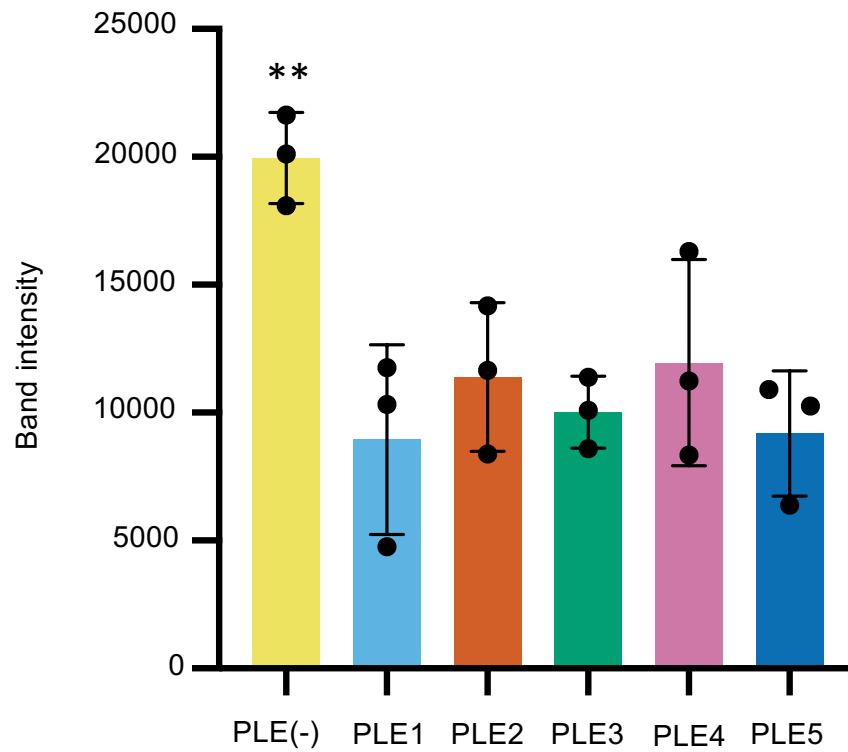

Supplement: FIG S8 [file mSystems.00358-20-sf008.pdf]
