# Supplementary material for: A Family of Viral Satellites Manipulates Invading Virus Gene Expression and Can Affect Cholera Toxin Mobilization
Source: mSystems. 2020 Oct 13;5(5):e00358-20. doi: 10.1128/mSystems.00358-20 (PMC7567579; doi:10.1128/mSystems.00358-20)
Supplement: FIG S3 [file mSystems.00358-20-sf003.pdf]

A

```
1  TTTTTTTTTTCTTACAACTTGAATAAAGTTGTTGATAATGATTCTCGTTTAATGCTATAA
2  -----TTTTTCTCACCACTTGAATAAAGTTGTTGATAATGATTCTCGTTTAATGCTATAA
5  ----TTTTCACTTATGACTTGAAAATAGTTGTTGATAATGATTCTCGTTTAATGCTATAA
3  ----TTTTCACTTATGACTTGAAAATAGTTGTTGATAATGATTCTCGTTTAATGCTATAA
4  ----TTTTCACTTATGACTTGAAAATAGTTGTTGATAATGATTCTCGTTTAATGCTATAA
        ***  ** *  ******* * **********************************

1  TTATATTGTAGGGGAGAGAATAAACCTTACCTACTTTTGATTATATGTTTCTCC-TACCT
2  TTATATTGTAGGG--GAGGA-AAACCTTACCTACTTTTGATTATATGTTTCTCC-TACCT
5  TTAATATGTAGGG--GAGGAT-AACCTTACCTACTTTTGATTATATGTTTCTCCTTACAT
3  TTAATATGTAGGGGAGAGAATAAACCTTACCTACGATTGATTATATGTTTCTCC-TACCT
4  TTAATATGTAGGGTGAAGGAAAAACCTTACCTACTTTTGATTATATGTTTCTCCTTACAT
   ***   *******   ** *  ************  ****************** *** *

1  TCATATTAT-CACACCTCTC-AATATTGCGCTGTCACGCAATT--AGCTAGCT--TGTAG
2  TCATATTAT-CACACCTCTC-AATATTGCGCTGTCACGCAATT--AGCTAGCT--TGTAG
5  TCATATAAT--ACACCTCTT-CAAATTGCGCTGTCACGCAATCTTAGCTAACTGCTATTG
3  TCATATATCAAACACCTCTCTTAAATTGCGCTGTCACGCAATC--AGCTAACTGCTATTG
4  TCATATAAT--ACACCTCTT-CAAATTGCGCTGTCACGCAATC--AGCTAACTGCTATTG
   ******     ********   * ******************   ***** **  * * *

1  CATACCCTACA--ATTTCCCTCAGTTCATAACCA-CGCTAGCGGTGGTGAGGGTTTCCTT
2  CATACCCTACA--ATTTCCCTCAGTTCATAACCA-CGCTAGCGGTGGTGAGGGTTTCCTT
5  CATACCCAATAGTATTCTCCCCTGTTCATAACCATTGTTAGC-GTGGTGAGGA-TTCCTT
3  CATACCCAATAGTATTTCCCTCAGTTCATGACCATTGTTAGC-GTGGTGAGGGTTTCCTT
4  CATACCCAATAGTATTTCCCTCAGTTCATGACCATTGTTAGCGGTGGTGAGGGTTTCCTT
   ******* * *  ***  ** * ****** ****  * **** *********  ******

1  ATGTTTGTTGTTATGTAAATTAATAAAGGTGGTTCCCTTCTTCAACCACTTTTATTTTTA
2  ATGTTTGTTGTTATGTAAATTAATAAAGGTGGTTCCCTTCTTCAACCACTTTTATTTTTA
5  ATGTTTGTTGTTATGTAAATTAATAAAGGTGGTTCCCTTCTTCAACCACTTTTATTTTTA
3  ATGTTTGTTGTTATGTAAATTAATAAAGGTGGTTCCCTTCTTCAACCACTTTTATTTTTA
4  ATGTTTGTTGTTATGTAAATTAATAAAGGTGGTTCCCTTCTTCAACCACTTTTATTTTTA
   ************************************************************

1  CCATAGCATTCAAGTCGTAAGAAAAAAAAAAA
2  CCATAGCATTCAAGTCGTAAGAAAAAAAAAAA
5  CCATAGCATTCAAGTCATAAG-----------
3  CCATAGCATTCAAGTCATAAGTGAAAA-----
4  CCATAGCATTCAAGTCATAAG-----------
   **************** ****
```

B

**ACATAACAACAAACATAAGGAAA**	Ideal PLE1 RNA target
**ACATAACAACAA**CA**ATAAGGAAA**	Upstream 12.1
TA**A**C**AACAA**AC**AACATAAGGAAA**	Upstream 5
C**C**TC**AACAACAAA**TT**TAAGGAAA**	Upstream16
**A**A**ATAACAACAAA**-**A**G**AAG**A**A**G**A**	Upstream 2
**A**ACC**AACAA**T**AA**TA**ATAAGG**AG**A**	Upstream 13
TA**ATAACAACAA**T**CAT**TGA**G**G**A**G	Upstream 7
**AC**TATTTT**AC**T**AA**T**ATAAGGAAA**	Upstream 12
